# Wood Pavement and Disinfectants

**Published:** 1895-07-20

**Authors:** 


### Wood Pavements and Disinfectants.

A new ailment is said to have been produced on rather an extensive scale, as a result of the action of the prolonged dry weather on the wood pavements of the London streets. The symptoms, which are very distressing, manifest themselves chiefly in the mucous membrane of the nose. The membrane becomes inflamed and thickened, the blood-vessels are turgid, and on very slight provocation there is bleeding to a considerable amount. The affection is said to be due to the millions of small particles of straw and other *débris* that lie in the wake of the vast horse traffic which from morn till midnight congests the leading London thoroughfares.

The subject is one quite worthy of investigation; and accordingly a visit was paid to the medical department of the London County Council at Spring Gardens, with a view of obtaining what information, if any, might be in the possession of Dr. Shirley Murphy and his assistants, and what steps, if any, the Council was taking to minimise the mischief. Last year the same, or a similar, ailment was complained of, and certain steps were then taken by the central and local authorities with the object of mitigating the danger. Those steps consisted almost entirely in flushing the principal thoroughfares well with plain water once or twice a day; and, in adding to the water a percentage of Condy's Fluid for those particular localities where the street *débris* seemed to be most offensive and dangerous. What the result of the flushing with disinfectants as compared with that of flushing with plain water might have been it was impossible to ascertain. The facts are, that in due time the complaints ceased to be made, and it was presumed that the cause had been removed.

It is said that the streets of London to-day, in the midst of one of the finest summers on record, "are in as bad or a worse condition than they often are in winter after a heavy snowstorm." If this be so—and all of us can form some kind of judgment on the subject by carefully observing the streets along which we are passing and re-passing every day—then the complaints of the public are more than justified. That vast masses of dust rise up like clouds, and are dashed about with violence by the winds, it is quite obvious. That such clouds of dust must be highly charged with particles of an irritating and poisonous character is equally clear; and that an efficient system of street watering would wholly subdue such pestilent dust-clouds goes without saying. Why then is the thing not done? County Councils and local authorities do not consist of scientific experts, whose chief business it is to balance theory against theory, and to sit on a philosopher's chair holding out a critical balance which, by reason of its scientific superfineness, can never be deflected towards definite action either on the right hand or the left. To them belongs the humbler, but not less practical, responsibility of putting in operation the actual science of the times as it is understood. The science of the times believes in street watering and disinfectants. The public is under the impression that for lack of street watering and disinfectants it is put to suffering and expense which it ought by no means to be called upon to bear. Let the County Council and the local authorities deal with facts as they are, and with science as it is immediately understood. If, after all this is done, the public still suffers, we shall at least have the satisfaction of knowing that the public authorities have grappled manfully with their responsibilities, and that the best has been done for the general well-being which the science of the times has attained to.

